# Observations on the Efficacy of Cervical Cauterization in the Treatment of Epilepsy

**Published:** 1820-05

**Authors:** 


FOR THE LONDON MEDICAL AND PHYSICAL JOURNAL.

### *Observations on the Efficacy of Cervical Cauterization in the Treatment of Epilepsy.* By Professor HALLE.

SYNCIPITAL *cauterization*, formerly used in the treatment of epilepsy and other diseases of the brain, had fallen into discredit, by the unfortunate results from it in the cases of DE HAEN and POUTEAU. This discredit has been considerably obviated by M. PERCY, in his *Pyrotechnie Chirurgicale;* and M. GONDRET has re-established, by some good observations, the use of an operation, the unfortunate consequences of which should undoubtedly be attributed to some impropriety in the mode of practising it, to some error in the appreciation of the circumstances where it is indicated, or to neglect of the ready means of preventing the inconveniences which may sometimes result from it. Lately, M. PARISET has profited by his situation in one of the hospitals which presents the most frequent favourable occasions for putting it to the test, to ascertain the utility of this remedy. I would, however, propose a comparison between this operation and that which I have employed for five-and-twenty years past in the same malady, which is similar to that employed by POTT in disease of the spine. It consists in the application either of two pieces of moxa or of actual cauteries on each side of the spinous processes of the cervical vertebræ, one of them near the occiput, the other near the commencement of the dorsal vertebræ. It was on the following occasion that I was led to adopt the use of this method.

I had come under my care a young man, who, besides frequent attacks of epilepsy, was affected with a chronic soporous affection, which continued to increase, and seemed to show that serous effusion was going on within the cranium. He was of a lymphatic, lax, indolent, and inactive, constitution. I had two issues made in the way above designated; my object being merely the relief of the soporous affection. It was soon removed, and, besides this, the epilepsy has entirely disappeared.

This success led me to employ the same mean in other cases of epilepsy, not complicated with soporous affections; and I have almost constantly obtained sufficient success to lead me to adopt this treatment, in preference to almost all the other measures commonly resorted to.

I should like an experimental comparison to be made between this method and the syncipital cauterization, and that it should also be compared with the other reputed anti-epileptic remedies; for I have also derived advantage from the internal use of the nitrate of silver. It appears to me, that, supposing the syncipital cauterization has sometimes been attended with the inconveniences which have been attributed to it, these need not be feared in the method I propose, after having myself put it to the test of experience. The accidents from the use of the nitrate of silver are much more to be feared; for this medicine often injures the mucous surfaces, producing ulcers all over the fauces, and still more dangerous ones in the mucous membrane of the stomach, as I and several of my colleagues have had occasion to observe. These accidents may, undoubtedly, be avoided, but much attention and constant watching are requisite for this; and the dangers are often such, that it becomes necessary to abandon the remedy. However, I should state, that, even in the use of the *cervical cauterization*, as I propose it, it has occurred to me to see a violent irritation extend over the occipital muscles, so that it became impossible for the measure to be borne beyond four or five months, notwithstanding the success obtained from it. Three months had indeed, in this case, elapsed without any attack of the epilepsy, which had occurred nearly every eighth day before the remedy was employed. On ceasing to preserve the issues, the paroxysms began to become frequent; but they at length gave way to the nitrate of silver, cautiously employed.—*Nouveau Journal de Médecine, tome* v.

